# An apple a day: an outbreak of cryptosporidiosis in Norway associated with self-pressed apple juice

**DOI:** 10.1017/S0950268819000232

**Published:** 2019-03-11

**Authors:** L. J. Robertson, T. T. Temesgen, K. R. Tysnes, J. E. Eikås

**Affiliations:** 1 Parasitology Lab., Department of Food Safety and Infection Biology, Faculty of Veterinary Medicine, Norwegian University of Life Sciences, PO Box 369 sentrum, 0102 Oslo, Norway; 2 Smittevernlege, Postboks 184, Fjell kommune, 5342 Straume, Norway

**Keywords:** *Cryptosporidium*, food-borne infections, gastrointestinal infections, outbreaks

## Abstract

In the autumn of 2018, an outbreak of cryptosporidiosis affected adult employees from the same company in Western Norway. The organism was *Cryptosporidium parvum*, GP60 subtype IIaA14G1R1. All those infected had drunk from the same container of self-pressed apple juice. Incubation period (1 week) and clinical signs were similar among those infected, although some experienced a more prolonged duration of symptoms (up to 2–3 weeks) than others. The infections resulted after consumption from only one of 40 containers of juice and not from any of the other containers. It seems that although *Cryptosporidium* oocysts were detected in a sample from another container, the contamination did not affect the whole batch. This is perhaps indicative of a restricted contamination event, either from contaminated ground in the orchard, or during collection of the fruit, or during processing. Although outbreaks of food-borne cryptosporidiosis have previously been associated with consumption of contaminated apple juice, most of the more recent outbreaks of food-borne cryptosporidiosis have been associated with salad vegetables or herbs. This outbreak, the first outside USA reported to be associated with apple juice, is a timely reminder that such juice is a suitable transmission vehicle for *Cryptosporidium* oocysts, and that appropriate hygienic measures are essential in the production of such juice, including artisanal (non-commercial) production.

On Monday 15 October 2018, six employees at the same work unit in Western Norway reported absence due to illness. All had similar symptoms, as follows: violent diarrhoea, abdominal pain with severe colic, cough, pain in the thighs, pelvis and back and exhaustion and fatigue. Cryptosporidiosis was diagnosed in a number of individuals via samples provided to their general practitioners. It was rapidly apparent that all those who were sick had partaken of some freshly pressed apple juice that had been brought into the workplace the previous Monday (8 October) by one of those who were sick. The apple juice had been made by the employee herself during the previous weekend, with apples picked in the orchard of her family's farm, and had been pressed at a neighbouring farm. The 5-L container of apple juice brought into the workplace had been left in a communal area for everyone who wished to help themselves. Retrospective questioning of the employees identified seven who had enjoyed cups of juice from the container. All six cases were among those who had drunk the apple juice. In all of those infected, the diarrhoea and other symptoms continued for at least 1 week, before gradually subsiding; for some individuals, the symptoms persisted for up to 2–3 weeks.

*Cryptosporidium* has been identified as a potentially food-borne parasite of importance in Europe [1], although a recent report from the European Food Safety Authority (EFSA) concluded that the relative importance of food as a transmission route, as compared with other vehicles of infection, is unknown [2]. Symptomatic infection (cryptosporidiosis) is characterised by those symptoms experienced in this outbreak, with the predominant sign being voluminous watery diarrhoea; abdominal pain, nausea, mild fever, anorexia, malaise, fatigue and weight loss are also common in cryptosporidiosis [2]. Although symptoms generally last for 2 to 3 weeks, sometimes longer, they resolve spontaneously in otherwise healthy people, but relapses are not uncommon. However, individuals with impaired immune systems are at risk of symptoms being more severe and protracted. This is important, as very few drugs are available for treatment of cryptosporidiosis, and nitazoxanide, a broad-spectrum antiparasitic and antiviral drug with proven success against cryptosporidiosis in otherwise healthy adults, is not licensed for use in Europe. Various global studies have shown that *Cryptosporidium* is a major cause of moderate-to-severe diarrhoeal disease in children younger than 5 years in some regions (sub-Saharan Africa and South East Asia). Although there are several species of *Cryptosporidium* that are infectious to humans, most infections are due to either *Cryptosporidium hominis* or *C. parvum*. The latter is zoonotic, and is a common cause of diarrhoeal disease in young ruminants, particularly calves. Based on data compiled in the EFSA Opinion [2], the first reported food-borne outbreaks of cryptosporidiosis (from 1983 to 2008) were often associated with unpasteurised dairy products or pressed apples (apple cider), such that of 14 outbreaks in this period, six were associated with dairy products and three with apple cider. However, in the last decade (from 2008 onwards) of the 10 reported outbreaks, none was associated with apple cider and only one with dairy products. Although, reports of food-borne cryptosporidiosis seem to be skewed towards Nordic countries [3], to date there have been no reports of cryptosporidiosis associated with either apple juice or dairy products from this region; all apple juice associated outbreaks have been reported from the USA, with the last reported outbreak in 2003 [4].

Although epidemiological inference strongly indicated that the apple juice was the source of this particular outbreak, further investigations were conducted to back up this supposition, and to try to shed light on how the apple juice had been contaminated.

On 2 November 2018, a faecal sample from one of the patients and a 1.5 L sample of apple juice (not from the same 5 L container that had been taken to the work place) were received at the Parasitology Laboratory, Norwegian University of Life Sciences, and analysed for *Cryptosporidium* oocysts. The apple juice was concentrated by centrifugation followed by immunomagnetic separation (IMS); the very small faecal sample was resuspended in 1.5 mL water and also concentrated by IMS (using a modified method based on that previously described for *Giardia* cysts [5]). Detection of *Cryptosporidium* oocysts in the concentrated samples was by immunofluorescent antibody staining (IFAT; Aqua-Glo, Waterborne Inc., New Orleans, USA), with 4′,6-diamidino-2-phenylindole used as an adjunct stain. Both the faecal sample and the apple juice sample were positive for *Cryptosporidium* oocysts. Only few oocysts were found in the faecal sample (as might be expected at the very end of an infection, for which symptoms had commenced over 2-weeks earlier), but large numbers (estimate of hundreds) of oocysts were found in the apple juice concentrate (after centrifugation and IMS), although many of them seemed distorted or broken. DNA was extracted from both the sample concentrates using the DNeasy^®^ PowerSoil^®^ kit (Qiagen, Norway) following the automated protocol IRT©V1 (QIAcube, Qiagen, Norway) in 50-μL elution volume. Nested polymerase chain reaction targeting the GP60 [6] and SSU-rRNA loci [7] was conducted, with sequencing of amplicons via a commercial company. Although DNA amplification from the apple juice sample was unsuccessful, the faecal sample was shown to contain *C. parvum* oocysts of subtype IIaA14G1R1. This is similar, but not identical, to a *C. parvum* subtype previously associated with two earlier cryptosporidiosis outbreaks in Norway; IIaA19G1R1 [8, 9], which were due to contact between schoolchildren and infected lambs and kids at a holiday farm. The same subtype has, however, been reported from faecal samples from calves in Poland [10] and Estonia [11], yaks in China [12], and humans in Slovenia [13] and Slovakia [14].

How the juice had become contaminated has not been clarified. The apples had been picked by hand from the trees (rather than collected from the ground), at a family farm by a group of family and friends, none of whom had any symptoms of illness, during the weekend. Animals had not been grazing around the trees, and the nearest livestock were sheep at a neighbouring farm. There were no young lambs in the flock at that time, and the sheep were retained behind an electric fence. The apples were collected into clean, dry containers and then driven to a farm in the neighbourhood for pressing. This farm presses apples for commercial production, as well as renting their equipment for individuals to press their own fruit; commercial production and pressing of apples by customers with their own fruit are not done in parallel. The pressing equipment is located in an enclosed area, not accessible by animals, and the equipment is washed between customers.

A total of around 200 L of apple juice was made, and as this was being produced it was collected into previously unused 5-L volume containers. These containers were divided amongst those who had been involved in picking and pressing the apples, and one of them ended in the work place where the infection occurred. However, there were no other reports of infections associated with the consumption of the apple juice in the other containers (another container was similarly shared with colleagues at another work place, without anyone becoming ill), indicating that the contamination did not extend to the entire 200 L, and was perhaps limited to only a couple of containers. This suggests that maybe only a very few apples used to make the juice had been contaminated. It was speculated that perhaps a couple may have been accidentally dropped during collection and then retrieved; if by misfortune they had landed in an area of high contamination, then there could have been localised high contamination in a very few apples. Although there was the possibility to wash the apples before pressing, spiking experiments have indicated that it is difficult to remove *Cryptosporidium* oocysts from the surfaces of apples; the most effective methods, such as rigorous manual washing in water with a detergent or by agitation in an orbital shaker with Tris-sodium dodecyl sulphate buffer, were found to remove less than 40% [15]. This experiment with spiked apples further indicated that *Cryptosporidium* oocysts on apple surfaces retain their infectivity for at least 4 weeks [15].

An alternative route of contamination of the apple juice could have been that the equipment was contaminated before use, and the oocysts were washed into the initial containers of apple juice. There is no evidence for the latter suggestion, and there were no reports of illness by others using the press. Nevertheless, the owner of the apple press reported the intention to implement use of a high-pressure hot water cleaner for use between apple lots and customers to assist in and improve the equipment cleaning process.

In conclusion, here we describe the first outbreak of apple juice-related cryptosporidiosis reported in Europe, and the first reported globally since 2003. Although the outbreak did not involve many people, it serves as a timely reminder that apple juice, and other beverages, may serve as efficient transmission vehicles for cryptosporidiosis.
